# Analysis of single nucleotide polymorphism in the promoter and protein expression of the chemokine Eotaxin-1 in colorectal cancer patients

**DOI:** 10.1186/1477-7819-5-84

**Published:** 2007-07-31

**Authors:** Dick Wågsäter, Sture Löfgren, Anders Hugander, Olaf Dienus, Jan Dimberg

**Affiliations:** 1 Atherosclerosis Research Unit, King Gustav V Research Institute, Department of Medicine, Karolinska Institute, Stockholm, Sweden; 2 Department of Clinical Microbiology, Ryhov County Hospital, Stockholm, Sweden; 3 Department of Surgery, Ryhov County Hospital, Stockholm, Sweden; 4 Natural Science and Biomedicine, University College of Health Sciences, Jönköping, Sweden

## Abstract

**Background:**

Previous studies suggest that chemokines (chemotactic cytokines) promote and regulate neoplastic progression including metastasis and angiogenesis. The chemokine eotaxin-1 is a powerful eosinophil attractant but also exerts chemotaxis of other leukocytes. Eotaxin-1 has been implicated in gastrointestinal disorders and may play an important role in colorectal mucosal immunity.

**Patients and methods:**

The objective of this study was to assess the role of eotaxin-1 in colorectal cancer (CRC). Levels of eotaxin-1 protein in CRC tissues (n = 86) and paired normal mucosa were compared after determination by ELISA. Plasma eotaxin-1 levels from CRC patients (n = 67) were also compared with controls (n = 103) using the same method. Moreover, a TaqMan system was used to evaluate the -384A>G eotaxin-1 gene variant in CRC patients (n = 241) and in a control group (n = 253).

**Results:**

Eotaxin-1 protein levels in colorectal tumours were significantly (P < 0.0001) higher than in normal tissue. Immunohistochemistry revealed eotaxin-1 expression in stromal cells such as fibroblasts and leukocytes of the CRC tissue. The plasma eotaxin-1 level in CRC patients was lower compared with controls (P < 0.0001). Patients with tumours classified as Dukes' stage B and C had lower levels than patients with tumours in Dukes' stage A. We found no difference in genotype distribution but noted a difference regarding allele distribution (P = 0.036) and a dominance of allele G in rectal cancer patients.

**Conclusion:**

The up-regulated eotaxin-1 protein expression in cancer tissue may reflect an eotaxin-1 mediated angiogenesis and/or a recruitment of leukocytes with potential antitumourigenic role. We noticed a dominance of the G allele in rectal cancer patients compared with colon cancer patients that was independent of eotaxin-1 expression.

## Background

Previous studies show that chemokines (chemotactic cytokines) are important factors promoting leukocyte attraction to sites of inflammation and cancer [1-3]. Some of the chemokines may promote and regulate neoplastic progression including metastasis and angiogenesis [4,5].

In the gastrointestinal tract the intestinal mucosa regulates trafficking of the leukocytes by chemokines such as CXCL5, CXCL8 and CXCL12 [6,7]. The chemokine eotaxin-1 (CCL11) is one member of the eotaxin gene family (eotaxin-1, eotaxin-2 and eotaxin-3) which is a group of eosinophil chemoattractants [8]. Eotaxin-1 binds exclusively to the receptor CCR3, which is present on eosinophils. Basophils, mast cells, Th2 cells, dendritic cells and endothelial cells also express CCR3 at variable levels [9-11]. Eosinophil accumulation in the peripheral blood and tissues is a marker of several diseases such as viral and parasitic infections, atopic disorders, eosinophilic gastroenteritis and pneumonia [12]. In the gastrointestinal tract, eotaxin-1 is expressed in mucosa and it has been suggested that eotaxin-1 has an important role in the maintenance of normal eosinophil homeostasis [13]. The expression of eotaxin-1 has been identified in macrophages, eosinophils, epithelial cells, endothelial cells, fibroblasts, malignant epithelial cells and smooth muscle cells [14-18]. Consequently, eosinophils seem to have the capacity to generate their own autocrine chemoattractant factor eotaxin-1 [19].

Eotaxin-1 may play a role in a number of chronic inflammatory diseases such as sinusitis, nasal polyposis, rhinitis, ulcerative colitis and other gastrointestinal disorders [9,20]. Increased plasma levels of eotaxin-1 have been shown in coronary artery disease and inflammatory bowel disease [21,22]. Moreover, it has been suggested that eotaxin-1 may protect against tumour progression by recruiting eosinophils with a capacity to release cytotoxic proteins [14].

Several single nucleotide polymorphisms (SNPs) of the eotaxin-1 gene have recently been described. One of these, the -384A>G variant, is located at the promoter region of the eotaxin-1 gene and has been shown to have functional effects on eotaxin-1 synthesis. This variant correlated significantly with an elevated plasma eotaxin-1 level in asthmatic patients. It has been postulated that the -384A>G locus may exert its influence through change in the eotaxin-1 gene transcription [23].

The aim of the present study was to investigate the relation of eotaxin-1 protein expression to, and the influence of the eotaxin-1 gene variant -384A>G on CRC.

## Materials and methods

This investigation was approved by the research ethical committee at the Faculty of Health Sciences, Linköping, Sweden (Dnr. 98113). Informed consent was obtained from each subject.

### DNA samples

Study materials comprised blood samples obtained from 241 patients undergoing surgical resections for primary colorectal adenocarcinomas at the Department of Surgery, Ryhov County Hospital, Jönköping, Sweden. The patient group represented 123 males and 118 females with a mean age of 70 years (range 29–93). All tumours were classified according to Dukes' classification system: stage A (n = 45), stage B (n = 102), stage C (n = 82), and stage D (n = 12). The tumours were localized in the colon (n = 121) and rectum (n = 120).

Blood donors (n = 253), from Ryhov County Hospital with no known CRC history and a normal blood status, were selected as controls. This group consisted of 133 males and 120 females, with a mean age of 68 years (range 50–83). All blood-controls came from the same geographical region as the CRC patients and all cases and controls were of Swedish Caucasian origin. All blood was stored frozen until DNA was extracted using QIAamp DNA blood kit (Qiagen, CA, USA).

### Plasma samples

Sixty-seven of the CRC patients and 103 blood-control donors were available for plasma collection. Patient blood was collected before surgery. All blood, including that from the control group, was separated by centrifugation within 1 h. Plasma was removed and stored at -70°C. The CRC patient group comprised 38 males and 29 females with a mean age of 69 years (range 29–88). The patient tumours were categorized according to Dukes' classification; stage A (n = 15), stage B (n = 29), and stage C (n = 23). Thirty-five tumours were located in the rectum and 32 in the colon. Controls consisted of plasma from 54 males and 49 females, with a mean age of 61 years (range 55–67).

### Tissue samples

Tissue samples available from 86 of the CRC patients were used in the study. The tumours came from 49 males and 37 females having a mean age of 69 years (range 29–88). Tumours were collected and classified according to Dukes' classification system: stage A (n = 16), stage B (n = 37), and stage C (n = 33). Tumours were localized in the colon (n = 43) and rectum (n = 43). Tumour tissue and adjacent normal mucosa (about 5 cm from the tumour) from each patient were excised and immediately frozen at -70°C until analysis.

Frozen tumour tissue and normal mucosa were thawed, homogenised in ice cold lysis buffer containing PBS (9.1 mM dibasic sodium phosphate, 1.7 mM monobasic sodium phosphate, 150 mM NaCl, pH = 7.4) and 1% Nonidet P-40, 0.5% sodium deoxycholate, 0.1% sodium dodecyl sulphate (SDS), 100 μg/ml phenylmethylsulphonyl flouride (PMSF), 2 μg/ml aprotinin, 1 mM sodium orthovanadate and 1 μg/ml leupeptin. The lysate was placed on ice for 30 minutes and then centrifuged at 13000 g for 10 minutes. Protein content of the supernatant fluid was determined for each sample using the Bradford protein assay (Bio-Rad Laboratories, UK).

### Eotaxin-1 genotype determination

DNA samples were genotyped using 5'-exonuclease allelic discrimination assay (Applied Biosystems, USA). TaqMan SNP genotyping assay was used for analysis of rs17809012 genotype (Applied Biosystems). Ten-to-fifteen ng DNA was amplified in a total volume of 12 μl containing 1× TaqMan Universal PCR master mix (Perkin-Elmer, Applied Biosystems), including 1× TaqMan SNP genotyping assays. Amplification was performed using an initial cycle at 95°C for 20 s followed by 40 cycles at 95°C for 3 s and 60°C for 30 s. A post-PCR endpoint plate read was performed on each plate using the 7500 Fast Real Time PCR system (Applied Biosystems). The manual calling option in the allelic discrimination application ABI Prism 7500 SDS software version 1.3.1 was then used to assign genotypes.

### ELISA of eotaxin-1

Eotaxin-1 in plasma and tissue was measured using a commercially available enzyme-linked immunosorbent assay (ELISA) kit (R&D Systems Europe, UK) following the manufacturer's instructions. The plasma eotaxin-1 concentration from CRC patients and control subjects was expressed as picograms per millilitre (pg/ml) and the eotaxin-1 protein levels of cancer and paired normal tissues from the CRC patients were expressed as picograms per milligram of protein (pg/mg).

### Immunohistochemistry of eotaxin-1

Fourteen tumour samples were available for immunohistochemical staining for determination of the cell type origin of eotaxin-1 expression. Staining was performed on 4 μm sections from formalin-fixed paraffin-embedded tissue blocks, using a standard protocol. Endogenous peroxidase activity was quenched by treatment with 3% hydrogen peroxide for 5 min. Sections were subsequently incubated with a primary mouse anti-human monoclonal CCL11/eotaxin-1 antibody (R&D Systems Europe, UK) in appropriate dilution overnight at 4°C. After rinsing in TRIS-buffered saline, sections were incubated with secondary biotinylated goat anti-mouse IgG (Santa Cruz Biotechnology, USA). Avidin-biotin peroxidase complexes (Dako Cytomation Denmark) were added, followed by visualization with 3,3'-diaminobenzidine tetrahydrochloride (Dako Cytomation). All sections were counterstained with Mayer's hematoxylin (Histolab Products AB, Sweden).

### Statistical analysis

Differences in the frequencies of the eotaxin-1 polymorphism between CRC patients and the control group and between clinical data within the CRC subgroup were analyzed using the Chi-square test. The Hardy-Weinberg equilibrium was tested for all polymorphisms. Differences in eotaxin-1 plasma levels between patients and control subjects were examined using the Mann-Whitney U test. Differences in eotaxin-1 protein expression between tumour and normal paired tissues were examined using the Wilcoxon signed rank test. Statistical analysis was performed using the SPSS for Windows computer package (Rel. 14.0, 2005, Chicago: SPSS Inc.). Results were considered significant at a level of P < 0.05.

## Results

### Eotaxin-1 genotype

To analyse the influence of eotaxin-1 on colorectal carcinogenesis, we investigated the prevalence of promoter -384A→G gene polymorphism in 241 CRC patients and 253 control subjects using the TaqMan system.

There was no significant difference in genotype distribution or in allelic frequencies, between CRC patients and control subjects (Table 1). When subdividing the patients in groups, colon (n = 121) versus rectum (n = 120), we noted a weak anomaly (P = 0.065) in genotype distribution (Table 2). However, we found a significant difference regarding allele distribution (P = 0.036). When assessing this difference we noticed a 51.7% (124/240) dominance of the G allele in rectal cancer patients compared with 42.1% (102/242) in colon cancer patients. When testing the allele distribution in colon and rectal cancer patients compared with control subjects respectively we found a dominance of the G allele in the rectal cancer patients (P = 0.036). No difference was seen in colon cancer patients compared with controls (P = 0.73). The genotype and allelic distributions in CRC patients and the control group were not associated with clinical characteristics such as age, gender, or Dukes' stage. All genotype distributions were in Hardy-Weinberg equilibrium.

**Table 1:** Genotypic and allelic distributions in % (n) of Eotaxin/CCL11 polymorphism in CRC patients and control subjects

**Genotype**	**CRC**	**Controls**	**Allele**	**CRC**	**Controls**
	**(n = 241)**	**(n = 253)**		**(n = 482 alleles)**	**(n = 506 alleles)**

- 384A →G					
A/A	27.0 (65)	30.8 (78)			
			A	53.1 (256)	56.5 (286)
A/G	52.3 (126)	51.4 (130)			
			G	46.9 (226)	43.5 (220)
G/G	20.7 (50)	17.8 (45)			

CRC patients vs. controls: Not significant

**Table 2:** Genotype and allele numbers of the Eotaxin/CCL11 gene polymorphism (- 384A → G) regarding to location and Dukes stage in CRC

	**Genotype**			**Allele**	
	**A/A**	**A/G**	**G/G**	**A**	**G**

Colon (n = 121)	37	66	18	140	102
Rectum (n = 120)	28	60	32	116	124

Colon vs. Rectum: Genotypes overall P = 0.065, Alleles P = 0.036

### Plasma levels of eotaxin-1

**Figure 1:**
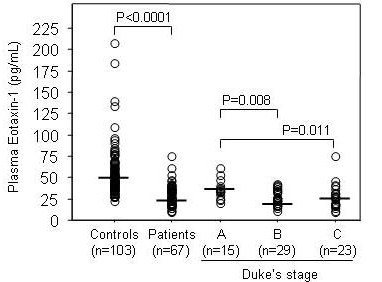
Plasma eotaxin-1 concentrations in healthy controls and CRC patients. CRC patients have significantly lower concentrations compared to controls. The plasma eotaxin-1 concentrations in Dukes' stage B and C patients were lower compared with Dukes' stage A patients. Medians are shown by horizontal bars.

Plasma levels of eotaxin-1 were measured by ELISA in 67 CRC patients and 103 healthy control subjects. The eotaxin-1 plasma concentration was lower (P < 0.0001) in CRC patients [median 26.2 (range 9.8–74.5) pg/ml] than in controls [median 48.9 (range 22.6–208) pg/ml] (Figure 1). A significantly lower eotaxin-1 plasma level was noted in Dukes' stages B (median 24.4 pg/ml) and C (median 25.6 pg/ml) as compared to Dukes' A (median 34.3 pg/ml) (P = 0.008 and P = 0.011, respectively) (Figure 1). The plasma levels of eotaxin-1 from CRC patients were not related to age, gender, tumour location or any eotaxin-1 allele/genotype investigated in this study (data not shown).

### Levels of eotaxin-1 in colorectal tissue

**Figure 2:**
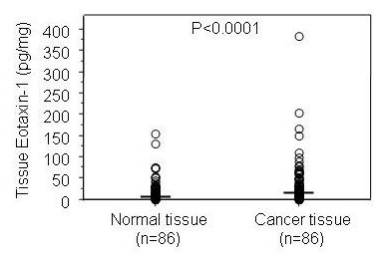
The eotaxin-1 protein level in cancer tissue was significantly higher compared to normal paired tissue. Medians are shown by horizontal bars.

Eotaxin-1 protein concentration was measured by ELISA in protein-lysates of colorectal cancerous tissues and matched normal tissues from 86 patients. The levels of eotaxin-1 protein in cancer tissue [median 17.4 (range 0–383) pg/mg] were higher when compared to normal tissue [median 9.7 (range 0–153) pg/mg] (P < 0.0001), (Figure 2). Evaluation of the relative expression (tumour vs. normal tissue) showed 74.4% (64/86) up-regulation. There was no correlation between levels of eotaxin-1 protein in analyzed tissue samples from the CRC patients and clinical characteristics such as age, gender, tumour location, Dukes' stage, or eotaxin-1 allele/genotype determined in this study (data not shown).

### Location of eotaxin-1 expression in CRC tissue

**Figure 3:**
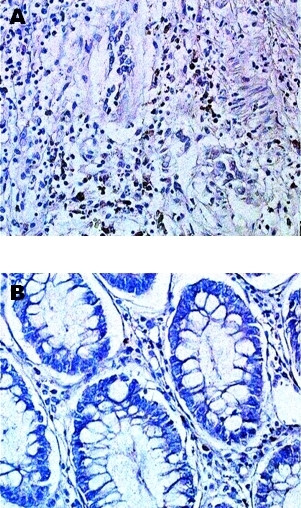
Images of immunohistochemical staining of eotaxin-1 in colorectal tissue from patients with CRC. Eotaxin-1 expressing stromal cells in cancer (A) and normal (B) tissue. Magnification, × 200.

Immunohistochemistry was performed to investigate the site of eotaxin-1 protein expression. Immunoreactivity showed heterogenous and focal staining of eotaxin-1 in tumour tissue and the resection border comprising normal tissue. The immunoreactivity was localised in stromal cells with morphological characteristics of fibroblasts, as well as leukocytes, including lymphocytes and macrophages (Figure 3). No staining was observed with secondary antibody exclusively (data not shown).

## Discussion

Chemokines which regulate leukocyte movement are suggested to play a crucial role in the congregation of lymphocytes in the intestine [6,7]. Eotaxin-1 is an eosinophil chemoattractant, expressed in the gastrointestinal mucosa by several stromal cells, which can also regulate chemotaxis of other cells such as basophils, Th2 lymphocytes and mast cells [8-10]. Eotaxin-1 seems to play a potential role in a number of diseases such as gastrointestinal disorders [9] and it has been suggested that eotaxin-1 could play a protective role against tumour progression [14], probably as a factor in tumour immune surveillance [22].

One SNP of the eotaxin-1 gene, -384A>G, has been showed to have a functional role in regulation of gene expression, and thus influences the eotaxin-1 synthesis. The presence of allele G is associated with higher plasma concentration of eotaxin-1 in asthmatic patients [23]. In our study, we focused on this SNP and showed that the genotype distribution and allelic frequency were not significantly associated with CRC patients as compared to controls. We did not detect any association between genotype and eotaxin-1 plasma concentration in plasma samples. However, we found a significant difference regarding allele distribution between rectal and colon cancer and noted a dominance of allele G in rectal cancer patients. In addition, we have demonstrated the distribution of the eotaxin-1 gene variant -384A>G in a Swedish population, for the first time.

Possible differences in the carcinogenesis of colonic and rectal carcinomas have been reported [24]. Hypothetically, different mechanisms may be involved in the cancer susceptibility in colon and rectum depending partly on allele G.

In diseases with immune and inflammatory response, such as Crohn's disease and Sjögren's syndrome, the circulating level of eotaxin-1 compared to controls is higher and lower, respectively [21,25]. In addition, a negative correlation between the circulating level of eotaxin-1 and eosinophil counts has been noted in Crohn's disease [21]. We found a significantly lower eotaxin-1 plasma level in CRC patients than in controls, and this difference increases with increasing Dukes' stage. Referring to the studies above [21,25], and our investigation, the findings indicate that there may be different mechanisms of immune regulation in different disease entities. Our study may show an imbalance in production of plasma eotaxin-1 from eosinophils or endothelial cells in CRC patients, and that a lower plasma eotaxin-1 level may reflect disease status of CRC. The discrepancy between tissue and plasma level ratio of eotaxin-1 in CRC may be explained by a biological difference that influences the secretion of eotaxin-1 in CRC tissue.

Using ELISA, we noted a significantly higher expression of eotaxin-1 protein in cancer tissue in comparison to matched normal tissue. Evaluation of the relative expression (tumour vs. normal tissue) showed 74.4% up-regulation. By using immunohistochemistry to determine the origin of the eotaxin-1 protein expression in CRC patient tissues we found immunoreactivity localised to stromal cells such as fibroblasts and leukocytes.

Very few reports have investigated eotaxin-1 expressing cells in relation to CRC outcome. Higher amount of eosinophilic infiltration of CRC has been reported to be a favourable prognostic indicator in CRC patients [26,27]. On the other hand, gastric carcinoma with eosinophilia correlates with unfavourable prognosis [28]. It has been suggested that eotaxin-1 directly mediates angiogenesis by CCR3^+ ^endothelial cells [11]. This effect may contribute to tumour growth and thereby poor prognosis in CRC. Thus, the role of eotaxin-1 and eosinophilia is not fully evaluated in CRC. We noted a significantly up-regulated eotaxin-1 level in cancer tissue which may reflect a potential eotaxin-1 mediated angiogenesis. Alternatively, this could mediate protection against tumour progress by recruitment of eosinophils and/or other CCR3^+ ^leukocytes with an antitumourigenic role.

The pathophysiological significance of the observed up-regulation remains unclear until basal mechanisms describing the transcriptional regulation and the signalling pathway of eotaxin-1 expression in CRC are elucidated. To date, little is known with regard to the influence of eotaxin-1 gene polymorphisms on the levels of eotaxin-1 in normal and neoplastic human tissues.

## Conclusion

We noticed a dominance of the G allele in rectal cancer patients compared with colon cancer patients that was independent of eotaxin-1 expression. The -384A>G eotaxin-1 gene variant may be partly involved in the pathogenesis of cancer in the rectum and colon. The up-regulated eotaxin-1 protein expression in cancer tissue represented by stromal cells may reflect an eotaxin-1 mediated angiogenesis and/or a recruitment of leukocytes with potential antitumourigenic effect. Finally, the plasma levels of eotaxin-1 were lower in CRC patients compared to controls, and the suppression seems to be more pronounced in severe disease stage.

## Competing interests

The author(s) declare that they have no competing interests.

## Authors' contributions

DW: Conceived the study, participated in its design, revised and helped in preparing the manuscript.

SL: Organized the laboratory work, revised and edited the manuscript.

AH: Surgeon who performed resection of the patient tissue and documented clinico-pathological characteristics.

OD: Carried out the genotyping and contributed to collection of the data.

JD: Performed the immunohistochemical study, the statistical analysis, the literature search, and prepared the manuscript.

All authors read and approved the final manuscript.
